# A novel iliotibial band and gluteus maximus tenodesis for the treatment of external coxa saltas in a patient with Ehlers–Danlos syndrome

**DOI:** 10.1093/jhps/hnv050

**Published:** 2015-07-23

**Authors:** Matthew C. Bessette, Raymond J. Kenney, Michael B. Geary, P. Christopher Cook, Brian D. Giordano

**Affiliations:** 1. Department of Orthopaedics and Rehabilitation, University of Rochester Medical Center, Rochester, NY 14642, USA; 2. School of Medicine, University of Rochester School of Medicine and Dentistry, Rochester, NY 14642, USA

## INTRODUCTION

We report a unique surgical treatment for external coxa saltans refractory to previous open and endoscopic management in a patient with Ehlers–Danlos syndrome. After failure of two endoscopic iliotibial band (ITB) lengthenings and one open gluteus maximus (GMax) lengthening, a novel procedure was conducted, which involved release of the GMax insertion and tenodesis of the anterior and posterior portions of the ITB to prevent pathologic translation over the trochanter.

## CASE REPORT

An 18-year-old female with Ehlers–Danlos syndrome (Body mass index 16.2 kg/m^2^) visited our institution with an 8-month history of debilitating ITB snapping that was unresponsive to prior attempts at non-operative treatment. On examination, she was globally hypermobile and had painful and reproducible snapping of the right ITB over the greater trochanter.

X-ray revealed normal femoroacetabular morphology, while magnetic resonance imaging showed thickening of the posterior one-third of the ITB. There was no evidence of intra-articular pathology.

After discussion of available treatment options, the patient elected to undergo index endoscopic Z-lengthening of the IT band. One year into an uncomplicated recovery, the patient began to experience painful recurrent snapping. A revision endoscopic ITB lengthening was conducted by creating a diamond-shaped window in the ITB. Unfortunately, the patient presented with recurrent symptoms after 5 months of initial relief and, at this time, open Z-lengthening of the GMax insertion was performed with direct open examination of the ITB and gluteal complex. After 18 months of pain-free function, the patient again experienced symptomatic recurrence. A more aggressive open tenodesis of the IT band and GMax release were proposed.

**Fig. 1.**
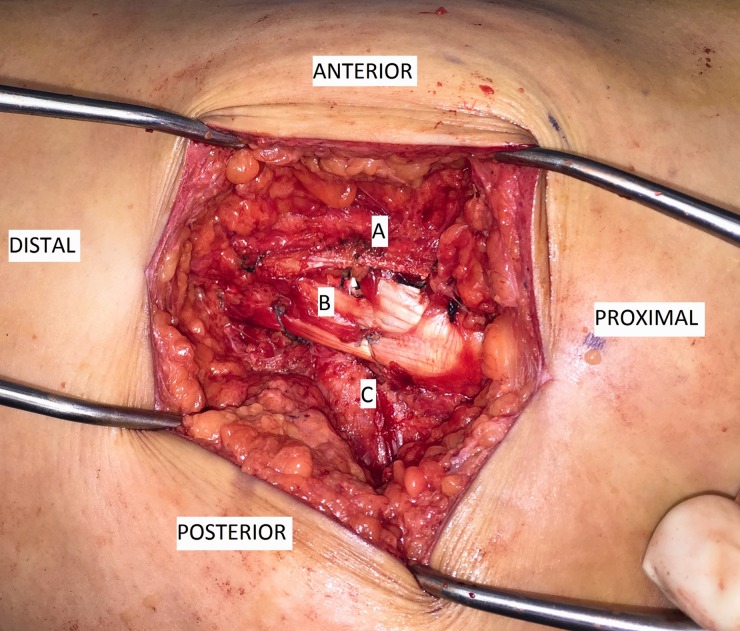
This picture illustrates the finished tenodesis viewed through a lateral incision. Structure A is the anterior portion of the divided IT band, which has been sutured to the anterior fibers of the vastus lateralis fascia and other local soft tissue. Structure B is the vastus lateralis, which has been partially sewn over the site of the posterior IT band and GMax tenodesis to the posterior peri-trochanteric femur. Structure C is the remaining visualized fascia of the posterior IT band and GMax fascia. The posterior sutures between Structures B and C originate from the suture anchors in the posterior femur.

For this novel procedure, the IT band was divided longitudinally and the GMax insertion was released. A trochantoplasty of posterosuperior facet was performed after examination revealed abrasion against the ITB. Suture anchors were placed proximal to the GMax insertion on the posterior border of the linea aspera and proximal femur (4.5 mm PEEK Corkscrew and 3.0 mm BioComposite SutureTak, Arthrex Inc, Naples, FL). The posterior division of the ITB and GMax was then secured using these anchors. The anterior fibers of the IT band were sutured to the anterior vastus lateralis and greater trochanter, as seen in Figure 1. Following this procedure, all pathologic subluxation of the ITB and GMax was eliminated.

At a minimum follow-up of 6 months, the patient did not experience symptomatic recurrence and had improved with respect to pain control and physical function.

## DISCUSSION

Symptomatic external coxa saltans is usually amenable to non-operative treatment. However, in rare cases, surgical intervention is warranted [1, 2]. External coxa saltans has been described in association with Ehlers–Danlos syndrome in the past [3].

Numerous techniques, both open and endoscopic, have been reported for the treatment of the external snapping hip. Refractory snapping that has not responded to prior surgical treatment presents a challenge to surgeons. Revision options are sparse, and there is a relative paucity of information regarding treatment for these patients who are typically young, active and demand a more functional lifestyle [4].

Our novel procedure limits pathologic translation of the IT band and GMax over the greater trochanter by statically anchoring the offending structures. For refractory cases of IT band or GMax snapping that has failed prior endoscopic and open interventions, GMax release with tenodesis of the IT band is a reasonable revision option that has demonstrated success in short-term outcomes.

## CONFLICT OF INTEREST STATEMENT

Brian Giordano is a paid consultant for Arthrex.
